# Dance experience sculpts aesthetic perception and related brain circuits

**DOI:** 10.1111/nyas.12634

**Published:** 2015-03-13

**Authors:** Louise P Kirsch, Kelvin Dawson, Emily S Cross

**Affiliations:** 1 Wales Institute for Cognitive Neuroscience, School of Psychology, Bangor UniversityBangor, North Wales, United Kingdom; 2 Department of Social and Cultural Psychology, Behavioural Science Institute, Donders Institute for Brain, Cognition, and Behaviour, Radboud UniversityNijmegen, The Netherlands

**Keywords:** action perception, affective judgment, emotion, aesthetics, fMRI, dance

## Abstract

Previous research on aesthetic preferences demonstrates that people are more likely to judge a stimulus as pleasing if it is familiar. Although general familiarity and liking are related, it is less clear how motor familiarity, or embodiment, relates to a viewer's aesthetic appraisal. This study directly compared how learning to embody an action impacts the neural response when watching and aesthetically evaluating the same action. Twenty-two participants trained for 4 days on dance sequences. Each day they physically rehearsed one set of sequences, passively watched a second set, listened to the music of a third set, and a fourth set remained untrained. Functional MRI was obtained prior to and immediately following the training period, as were affective and physical ability ratings for each dance sequence. This approach enabled precise comparison of self-report methods of embodiment with nonbiased, empirical measures of action performance. Results suggest that after experience, participants most enjoy watching those dance sequences they danced or observed. Moreover, brain regions involved in mediating the aesthetic response shift from subcortical regions associated with dopaminergic reward processing to posterior temporal regions involved in processing multisensory integration, emotion, and biological motion.

## Introduction

When watching a live performance of *Swan Lake*, an observer might already be familiar with Tchaikovsky's famous score, attended a previous performance of this ballet, or even performed some parts of the choreography during a childhood ballet class. Whether and how we have previously experienced an action has the potential to profoundly shape the brain's response during action observation. Action understanding is thought to be facilitated through the direct matching of observed actions onto one's own motor system via a motor-simulation mechanism.^1^–^5^ Several studies demonstrate increased brain activity in people watching familiar movements within sensorimotor brain regions collectively termed the action observation network (AON).^6^–^12^

Returning to the *Swan Lake* example, it is likely that an observer's aesthetic experience can also change depending on prior experience with the piece being observed. Investigation of aesthetic experience at brain and behavioral levels has given rise to the burgeoning field of neuroaesthetics, which seeks to quantify and characterize the relationship between neurobiology and aesthetic judgment. Nadal *et al*. describe aesthetic judgment as a fully embodied and enactive process in which expertise plays an important role.^13^ Authors note distinct neural substrates that subserve positive aesthetic judgments, including somatosensory cortical regions of the AON,^14^,^15^,^25^ subcortical reward circuitry,^16^–^18^ and areas of prefrontal cortex involved in top-down processing and evaluative judgments.^19^–^21^

Although general familiarity and liking appear to be related,^22^–^24^ it is less clear how motor familiarity, or action embodiment, impacts aesthetic appraisal. Cross *et al*. began to address this question by investigating the relationship between self-report motor ability and liking of professionally performed dance movements.^25^ In this fMRI study with nondancers as participants, they found that movements participants rated as most difficult to reproduce were also rated as most pleasing to observe, which in turn coincided with greater AON engagement. In a follow-up between-subjects training study, Kirsch *et al*. exposed participants with no previous dance experience to one of three training conditions: physical dance practice, audiovisual experience, or auditory experience only, all with the same dance music video stimuli.^26^ Prior to training, Kirsch *et al*. replicated the work of Cross *et al*. by demonstrating that participants liked movements more that were rated as more complex.^25^ After training, a positive correlation emerged among participants in the physical training group such that increased liking was associated with increased ability to perform a dance movement. This finding suggests that once an observer gains physical experience with a perceived (dance) movement, the relationship between liking and embodiment shifts to a positive direction, such that more executable movements are now more likable. How such experience changes neural representations of action remains unknown.

Here we conducted a within-subjects fMRI version of the study by Kirsch *et al*.^26^ that directly compared how learning to perform an action impacts brain responses when one is watching and aesthetically evaluating that action. Nondancers experienced a series of dance movements via three modalities: auditory only, auditory and visual, and auditory, visual, and motor. Our main question concerns how experience shapes affective judgment of newly learned actions. Our design enables evaluation of how increased experience with a particular movement changes associated affective judgment of this movement compared to unfamiliar dance sequences. Our central hypothesis is that increased experience with a previously unfamiliar action should increase liking and that such changes will be reflected by modulation of AON activity based on experience.

## Methods

### Participants

Twenty-two healthy right-handed adults with no prior dance or video game experience participated. The Bangor University School of Psychology Ethics Committee approved all components of this study. Four participants were excluded due to technical problems during scanning, yielding 18 participants for the final sample (10 females; mean age = 23.6 years, SD = 5.1 years).

### Stimuli and apparatus

Eight dance sequences from the game *Dance Central 2* (Harmonix Music Systems, Cambridge, MA, 2011) for the XBox 360 Kinect™ console were chosen that featured gender-neutral dance movements with minimal background motion and a medium level of difficulty (mean video length = 2:19 min). Sequences were randomly paired and assigned to one of the four training conditions: physical, visual, and auditory experience (PVA), visual and auditory experience (VA), auditory experience only (A), and no experience/untrained (UNT). A total of four different training groups were assembled, meaning that each pair of dance sequences was trained in all four training conditions across participants.

For fMRI, 64 dance segments were extracted from the eight full dance sequences (eight from each sequence; mean = 3.95 s). Each stimulus was edited to feature one complete dance move involving whole-body motion and significant spatial displacement of the limbs.^14^ To obtain a task-specific visual baseline, 10 extra stimuli of the avatar standing in place for 5 s were created.

### Behavioral training procedure

**Figure 1:**
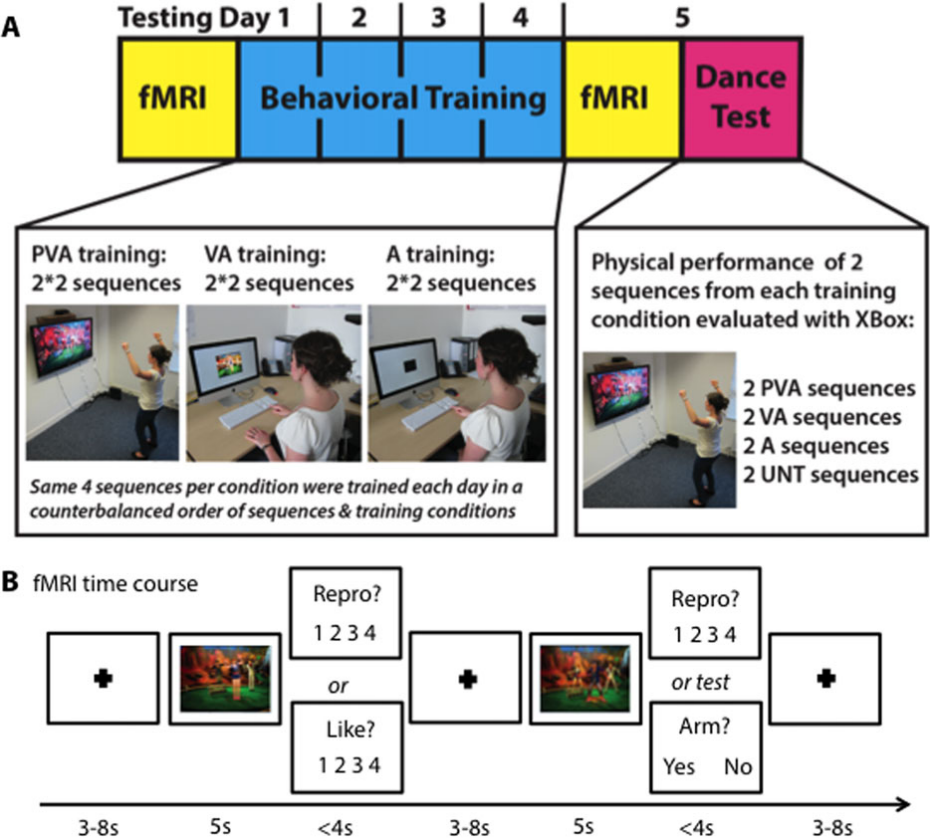
(A) Experimental design depicting the phases of the study in chronological order. All participants completed two identical fMRI sessions, four consecutive days of behavioral training, and a final dance test. Representation of the three training conditions: physical, visual, and auditory experience (PVA); visual and auditory experience (VA); auditory experience only (A). Participants learned two distinct sequences in each training condition but physically practiced (PVA), watched (VA), or listened (A) to each sequence twice on each of the 4 days of training (for the PVA and VA conditions, once with a male and once with a female avatar). For the dance test on day 5, participants performed all eight sequences once in a counterbalanced order. (B) Time course of the fMRI phase. Participants watched short sequences taken from the longer training videos. After a fixation screen (length pseudologarithmically randomized between 3 and 8 s), participants watched a 5-s dance movement and were aware that they would then have to answer a rating question concerning how much they liked watching the previous movement (“How much did you like the movement you just watched?” which was shortened to “Like?” in the actual experiment) or how well they could perform the movement just watched (“How well could you reproduce the movement you just watched?” which was shortened to “Repro?” in the actual experiment). Participants provided their responses via a four-button response box placed on their lap on which they rested the index finger and middle fingers of both hands over the buttons. The Likert-scale ranged from 1 (not at all) to 4 (extremely). The question remained on the screen until participants responded or for a maximum of 4 seconds. In one run, 10 additional video stimuli featuring the main dancer standing still were presented throughout the functional runs and required no response. Finally, six additional videos were included for attentional control questions. After each one of these control trials, participants were asked a question that required a “yes” or “no” response (“Did the dancer place at least one arm above her head?”). This was designed to ensure that the participants paid full attention to the dancer's movement in each stimulus.

Participants were randomly assigned to one of four training groups in which they experienced the same pairs of sequences assigned to the three training conditions (PVA, VA, and A) across four consecutive days of training (Fig.1A). The order in which participants completed the training conditions was counterbalanced within and between participants across training days. For more details about the training conditions, see supplementary methods in Supporting Information (these training conditions are also from Kirsch and Cross, unpublished data).

### Posttraining performance assessment

On the final testing day, participants performed the six sequences used in training as well as two additional untrained sequences. The test followed the same paradigm as the PVA training phase of the study: participants physically performed all eight dance sequences by mirroring the avatar's dance movements while the Kinect™ system captured and scored their movements.

### Neuroimaging procedure

During identical pre- and posttraining fMRI sessions, participants completed two runs containing 80 trials each (64 stimuli, 6 attentional test videos, and 10 still-body sequences). All stimuli were novel to participants during the pretraining fMRI scan. Each video was followed by one of two questions that required participants to aesthetically rate the observed dance movement (“How much did you *like* the movement you just watched?”) or assess their physical ability to reproduce the movement (“How well could you *reproduce* the movement you just watched?”; see Fig.1B).

The experiment was carried out in a 3T Philips MRI scanner using a SENSE phased-array head coil. For functional imaging, a single-shot echo planar imaging sequence was used (T2*-weighted, gradient echo sequence; echo time TE = 30 ms; flip angle, 90°). The scanning parameters were as follows: repetition time TR = 2000 ms; 30 axial slices; voxel dimensions, 3 mm^3^ with voxel slice thickness of 4 mm and slice gap of 0.8 mm; field of view (FOV), 230 × 230 × 143 mm^3^; matrix size, 128 mm^2^; anterior–posterior phase encoding. Parameters for T1-weighted anatomical scans were 240 mm^2^ matrix; voxel dimensions = 2 mm^3^; TR = 12 ms; TE = 3.5 ms; and flip angle = 8°. For each run of each scanning session, the first two brain volumes were discarded to reduce saturation effects.

### Behavioral training analysis

Raw numeric scores recorded by the Kinect™ system each day of PVA training for each participant were used to quantify participants’ performance across the training days and test day. Details of analysis and results for physical performance and VA recognition accuracy are detailed in supplementary results 1 in Supporting Information.

### Posttraining behavioral test

Raw scores were averaged within training conditions to produce an average score per participant for each of the four test conditions. A repeated-measures ANOVA on these scores evaluated the impact of different kinds of experience on physical performance. Pairwise comparisons (Bonferonni-corrected) subsequently evaluated any differences between conditions in more detail. Degrees of freedom reflect the Greenhouse–Geisser correction where sphericity has been violated.

### Liking and reproducibility judgments

To evaluate the impact of different kinds of experience on participants’ ratings of movement liking and reproducibility, ratings for stimuli from each training condition were averaged for each participant for each rating session. Repeated-measures ANOVAs were conducted on the averaged liking and reproducibility ratings, with rating session (pre-/posttraining) and training type as factors. We then ran paired *t*-tests to evaluate pre- and posttraining differences for each training condition. We also conducted a correlation analysis between both ratings, before and after training, by training condition, to assess the hypothesized relationship between reproducibility and liking.

### Reproducibility ratings and physical performance

To evaluate the relationship between objective (performance scores) and subjective (ratings) ability to reproduce a movement, we conducted a series of correlations taking into account training type and learning stage (pre-/posttraining).

### fMRI data analysis

Neuroimaging data from each scanning session were first analyzed separately. Data were realigned and unwarped in SPM8 and normalized to the Montreal Neurological Institute (MNI) template with a resolution of 3 mm^3^. Slice timing correction was performed after realignment. Functional data were normalized to individual participants’ T1 anatomical scans with a resolution of 3 mm^3^. All images were then spatially smoothed (8 mm). A design matrix was fitted for each participant, with each type of dance video, as well as button presses, attentional control videos, still-body videos, and fixation modeled as a boxcar function convolved with the standard hemodynamic response function.

### Action observation network mask

**Figure 2:**
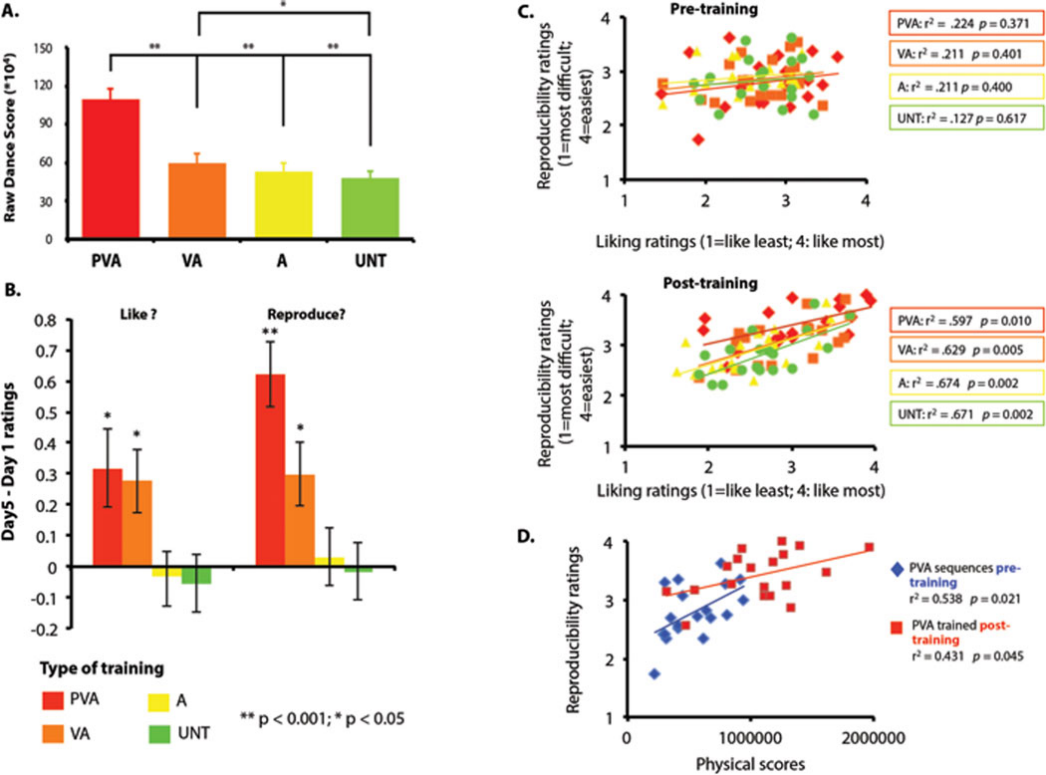
(A) Mean dance scores for all the sequences performed during the dance test on day 5 for each training condition. Significant differences between the PVA and other conditions were found, as well as a difference between the VA and UNT conditions. PVA, physical + visual + audio training; VA, visual + audio training; A, audio-only training; UNT, untrained sequences. **P < 0.001 and *P < 0.05. (B) Differences between pre- and posttraining ratings, for questions concerning liking and reproducibility. *P < 0.05; **P < 0.001. (C) Relationship between reproducibility and liking ratings, pre- and posttraining, for all training conditions. The right-side panel indicates the Pearson correlation factor and *P* value for each training condition. (D) Relationship between subjective and objective physical scores, illustrated by correlations between reproducibility ratings and physical performance scores pre- and posttraining for PVA sequences. The panel on the right side of the plots specifies the Pearson correlation factor and *P* value for the PVA training condition pre- and posttraining.

The first group-level analysis evaluated brain regions that were more active when observing a dancer's body in motion versus standing still on day 1. Such a contrast enables the localization of brain regions responsive to dance per se and not extraneous features that are not of interest for this study (e.g., dancers’ identity, background). Regions that emerged from this contrast, illustrated in Figure2A, created a task-specific mask for all subsequent analyses reported in this paper, at the *P* < 0.01, *k* = 10 voxel level.

The main neuroimaging analyses were designed to achieve the following three objectives:

#### Aesthetic evaluation of novel dance movements

A parametric analysis was run on the pretraining data, including individual participants’ liking ratings for each movement sequence as a parametric regressor. All conditions are considered together in this analysis, as they have yet to be trained and are equally novel at this stage.

#### Interaction between amount of experience and liking

We next ran the same analysis on the posttraining data. In this analysis stimuli of all conditions were also collapsed in a one-column matrix, as behavioral results showed a gradual increase of physical performance from UNT to A to VA to PVA conditions, and the same pattern was reflected in liking scores. This analysis should therefore reveal how an increase in experience and liking modulates brain activity and to some extent reveals how experience and liking interact.

#### Interaction between pre- and posttraining scans with increased liking

The final imaging analyses evaluated the impact of training with increased liking across scan session by calculating scan session by training interactions for the previous two analyses, in both directions (day 1 > day 5 and day 5 > day 1).

All neuroimaging analyses were evaluated within the task-specific mask with a voxel-wise threshold of *P* < 0.001 uncorrected and *k* = 10 voxels. Table1 lists all brain regions that emerge from these analyses, with FWE-corrected activations at the *P* < 0.05 threshold denoted with bold font. Anatomical localizations were assigned based on consultation of the Anatomy Toolbox^27^,^28^ in combination with the SumsDB online search tool (http://sumsdb.wustl.edu/sums/).

## Results

### Behavioral results

#### Training performance

Participants’ performance improved across training days (supplementary results 1 in Supporting Information). Analysis of performance scores from the dance test on day 5 revealed a significant effect of training condition on dance score (*F*_3,57_ = 77.861, *P* < 0.001), whereby participants performed sequences they physically practiced (PVA condition) significantly better than sequences from every other training condition (all *P* values < 0.001) and performed VA sequences significantly better than untrained sequences *(P* = 0.037). No other differences between training conditions reached significance (Fig.2A).

#### Liking and reproducibility ratings

To test the hypothesis that experience impacts perception of movement feasibility and liking, we evaluated the impact of training experience on both ratings, pre- and posttraining. Different kinds of training experience did indeed significantly impact participants’ ratings of their ability to reproduce the observed movements (*F*_3,51_ = 6.302, *P* = 0.001) as well as their affective judgments of movement (*F*_1,672,28.419_ = 7.9, *P* = 0.003) with significant interactions between training type and time of ratings, respectively, for reproducibility and liking (*F*_2.165, 36.798_ = 19.165, *P* < 0.001; *F*_3,51_ = 7.208, *P* < 0.001) (Fig.2B). Paired *t*-tests revealed significant effects of training on both questions for PVA and VA experience: PVA-repro, *t*(17) = −243, *P* = 0.027; VA-repro, *t*(17) = −2.784; PVA-like, *t*(17) = −5.728, *P* < 0.001; VA-like, *t*(17) = −2.830, *P* = 0.012 (for differences between training conditions, see supplementary results 2 in Supporting Information). These results broadly mirror what we find with performance on the dance test on day 5; participants performed better, judged they could reproduce better, and liked more movements for which they physically and/or audiovisually trained.

To further explore the relationship between liking and reproducibility, we evaluated correlations between ratings for each question from each training condition, pre- and posttraining. As Figure2C illustrates, a significant positive correlation emerged between participants’ ratings for how much they enjoyed watching the movements and how well they could reproduce them *only* posttraining, and for *all* training conditions. This suggests that for these particular stimuli, some degree of experience with this general type of stimulus is required for the positive relationship between aesthetic value and perceived embodiment to emerge. Most importantly, and replicating what was found in an earlier between-subjects study using a similar paradigm,^26^ participants liked movements more the better they thought they could reproduce them.

To explore the relationship between objective and subjective ratings of physical ability and how these might change with experience, we ran correlations between physical scores and reproducibility ratings pre- and posttraining. On the first day a moderate correlation emerged between day-1 dance scores and reproducibility ratings given to the same sequences pretraining (Fig.2D). This relationship persisted on day 5, although somewhat attenuated. Thus, it appears that subjective judgment of reproducibility correlates well with objective physical scores and does not disappear with increased physical experience.

### fMRI results

#### AON mask

**Figure 3:**
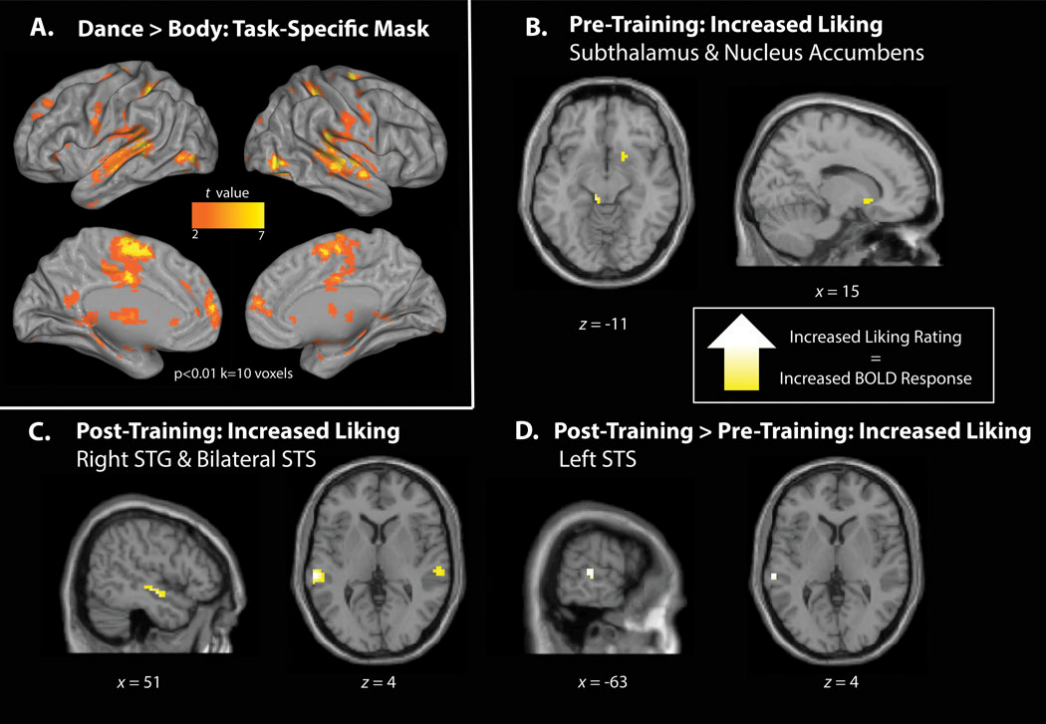
(A) Control dance movements > still-body contrast at *P* < 0.01, *k* = 10 voxels, used as mask for all subsequent contrasts. (B) Pretraining parametric analysis of all training conditions with increasing liking ratings. Regions whose response magnitude increases the more participants enjoyed watching movements after the week of training (from all four training conditions) included the right superior temporal gyrus (STG) and bilateral superior temporal sulci (STS). (C) Posttraining parametric analysis of all training conditions with increasing liking after training. Shown are regions activated the more a participant likes an observed movement that s/he has either PVA, VA, or A or UNT experience with. (D) Interaction between parametric effects of liking from post- and pretraining scan sessions. This analysis shows regions that are activated the more highly likable an observer rates an observed movement after 1 week of experience compared to before training.

In this study we focus our analyses within a mask of brain regions specific to watching whole-body movement. We created this mask by comparing attentional test videos that showed extra untrained sequences and still-body sequences on day-1 scans. This analysis yielded broad activation of the AON, consistent with results by Cross *et al*.^25^ from a similar analysis. All subsequent analyses were run within this task- and sample-specific mask (Fig.3A).

#### Aesthetic evaluation of novel dance sequences

To explore the impact of positive affective evaluation on brain activity while watching dance, we ran a parametric analysis on day-1 scans, taking individual participants’ ratings of how much they liked each movement as parametric regressors. Searching within the AON mask described above revealed small clusters of activity within the subthalamic nucleus and nucleus accumbens (Fig.3B; Table1a). The inverse contrast, evaluating brain regions becoming more active the *less* an observed movement is liked, did not reveal any suprathreshold activations.

**Table 1:** Regions associated with an increase in liking, depending on experience, with AON mask

		MNI coordinates			Putative		Cluster	*P*_corr_
Region	BA	*x*	*y*	*z*	functional name	*t* value	size	value
(a) Increase liking pretraining								
L subthalamus		−6	−31	−14		5.46	16	0.239
R Nucleus accumbens		15	14	−8	NAcc	4.02	15	0.251
R Nucleus accumbens		18	8	−14		3.94		
(b) Increase liking posttraining, increase experience								
**L superior temporal sulcus**		−60	−28	4	STG	5.57	83	0.017
R middle temporal gyrus		51	−16	−8	MTG	4.62	22	0.179
R superior temporal sulcus		63	−25	4	STG	4.24	24	0.163
(c) Increase liking, day 1 > day 5								
No suprathreshold cluster								
(d) Increase liking, day 5 > day 1								
L superior temporal sulcus		−63	−28	4	STG	4.04	11	0.805

Note

NAcc, nucleus accumbens; STG, superior temporal gyrus; MTG, middle temporal gyrus. All results at *P* < 0.001 uncorrected, *k* = 10 voxels; bold regions correspond to FWE-cluster corrected regions.

#### Aesthetic evaluation of trained dance sequence: interaction between experience and aesthetic evaluation of an observed movement

To explore the effect of liking experienced dance movements via different modalities, we ran a similar parametric analysis on the posttraining fMRI data. As behavioral results revealed a gradual increase of physical performance from UNT to PVA (Fig.2A), as well as for liking (Fig.2B), this analysis should inform how modulation of physical ability and liking by different kinds of experience is related to brain activity. This contrast revealed activity in bilateral superior temporal gyri (STG) and right middle temporal gyrus (Fig.3C; Table1b). The inverse contrast did not yield any suprathreshold activations.

#### Interactions between pre- and posttraining scans with increased liking: day 1 > day 5 and day 5 > day 1

The final imaging analyses provided the most rigorous test of how training experience impacts brain activity when subjects are viewing aesthetically pleasing movements by comparing parametric effects of increased liking between pre- and posttraining scans. To test whether any AON regions were more responsive when viewing liked movements when all movements were novel and untrained (prescanning session) compared to after the dance stimuli had been seen, listened to, and practiced, we first compared pretraining > posttraining parametric analyses. This analysis did not reveal any suprathreshold activations. However, when we evaluated whether any regions within the AON show a greater response to increased liking in the posttraining scan compared to the pretraining scan, the left STS emerged (Fig.3D; Table1d). This suggests that the left STS is sensitive to movements that are aesthetically pleasing to watch only after these movements have been experienced.

## Discussion

The main objective of this study was to explore how affective judgment, physical ability, and perceived ability to reproduce a dance movement relate at behavioral and brain levels. We found not only that training positively impacted liking and reproducibility ratings but also that specific parts of the AON are sensitive to such training experience when one is observing aesthetically pleasing movements. Implications of these findings are considered in turn.

### Interactions among experience, liking, and reproducibility

The behavioral data show that PVA and VA significantly experience shape perception. Similar to what Kirsch *et al*. found using a between-subjects design,^26^ participants reported increased liking and perceived ability ratings for movements they physically practiced or passively observed across four consecutive days. This suggests that even a small amount of sensory experience with a movement sequence, such as watching it while listening to its accompanying soundtrack, shapes perception in that these movements were liked more and performed better. It is instructive to consider this result together with Cross *et al*.'s finding of a negative relationship between liking and perceived ability for movements that participants had never performed.^25^ The finding that, after training, liking and reproducibility ratings are positively correlated (Fig.2D, bottom panel) is understandable in light of how experience shapes perceptual fluency.^29^,^30^ What is less consistent with previous work is the lack of negative correlation between liking and perceived ability pretraining (Fig.2C, top panel). This discrepancy could be due to differences in stimuli (classical ballet in the Cross *et al*. study^25^ versus street dance moves set to pop music in this study) and to the fact that, even before training, some of the movements in this study might have been more visually or physically familiar to participants than those used by Cross and colleagues.^25^

Another novel contribution offered by the behavioral data is that participants’ subjective evaluations of their dance ability were generally accurate, as evidenced by the positive correlation between subjective and objective dance ability scores (Fig.2D). This is a potentially useful validation for future studies interested in using self-assessment ratings of ability to perform complex actions.

### The impact of liking and experience on AON activity

During the pretraining scan, all movements were equally unfamiliar to participants. As a result, the parametric analysis of increased liking ratings for dance stimuli in the pretraining scan revealed regions within the AON mask most engaged when viewing the most aesthetically pleasing novel movements. This analysis revealed increased activity in the nucleus accumbens and parts of the thalamus. Dopaminergic neurons within the nucleus accumbens play a critical role in reward processing, and past work finds evidence for this brain region's engagement when participants view pleasant or reinforcing visual stimuli^31^,^32^ or listen to music.^33^ Moreover, Chatterjee and Vartanian discuss a role for the nucleus accumbens in more generalized reward processing during aesthetically pleasing experiences,^20^ an interpretation consistent with the present findings.

After training, and from the scanning session × liking parameter interaction, we found that regions associated with increased liking shifted to lateral/temporal cortices, as bilateral superior temporal and right middle temporal gyri showed increased responses with higher liking scores. As well as being critically involved in the recognition of biological motion,^34^,^35^ STS is associated with multisensory integrative processes. Moreover, Chen *et al*. suggest a close association between musical rhythm perception and movement coordination within STG and identify it as an important node for facilitating auditory–motor interaction in the context of rhythm.^37^ STS also plays a role in emotion.^38^–^40^ In one recent study Grèzes *et al*. have demonstrated structural connections between STS and the amygdala, a subcortical brain region implicated in emotional processing.^40^ In this study, the left STG in particular emerges as the core region influenced by both sensorimotor experience and increased liking, when we control for pretraining activity. Although speculative at this stage, its increased engagement following training might reflect a binding of auditory, visual, and motor experience to produce a more pleasurable and emotional experience for the perceiver.

To conclude, this study is the first to investigate how different kinds of sensorimotor experience impact aesthetic ratings and neural responses to watching dance. The more modalities through which an observer has experienced a movement, the more enjoyment the observer derives from watching that movement. Moreover, after such experience has been acquired, brain regions involved in mediating the aesthetic response shift from subcortical regions associated with dopaminergic reward processing to posterior temporal regions involved in multisensory integration, emotion, and biological motion processing. These findings have the potential to inform a number of domains beyond neuroaesthetics, including arts education, choreographic practice, and marketing.

## Supporting Information

**Supplementary methods**. Description of the procedure for the different training conditions: physical, visual, and auditory (PVA) experience; visual and auditory (VA) experience; and auditory experience only. **Supplementary results 1**. Physical performance over the 4 days of training and VA accuracy. **Supplementary results 2**. Affective judgment—difference between training conditions.
