# Supplementary material for: Dance experience sculpts aesthetic perception and related brain circuits
Source: Ann N Y Acad Sci. 2015 Mar 13;1337(1):130–9. doi: 10.1111/nyas.12634 (PMC4402020; doi:10.1111/nyas.12634)
Supplement: Supplementary file 1 — Supplementary methods. Description of the procedure for the different training conditions: physical, visual, and auditory (PVA) experience; visual and auditory (VA) experience; and auditory experience only. Supplementary results 1. Physical performance over the 4 days of training and VA accuracy. Supplementary results 2. Affective judgment—difference between training conditions. [file nyas1337-0130-sd1.docx]

**Supplementary information:**

1. SUPPLEMENTARY METHODS

***Physical + Visual + Auditory Experience (PVA).***

For sequences participants physically practiced, they stood approximately 2 meters away from a 52” Sharp flat screen television mounted on the wall in front of them. Participants’ task was to mirror the dance movements of the avatar in the 'Dance Central 2' Xbox 360 game and concentrate on improving their performance during subsequent sessions. The Kinect™ motion capture system assessed participants' movements compared to the avatar’s movements and assigned a score based on accuracy of mirroring the avatar. Participants received on-screen feedback about how well they were performing in the form of a final score after each sequence. Participants’ dance scores were recorded by the researcher and used as the objective measure of dance performance for the behavioural analyses.

***Visual + Auditory Experience (VA).***

For the sequences for which participants acquired visual and auditory experience, they sat comfortably in front of a computer running Psychophysics Toolbox 3 in MATLAB R2010a (Mathworks Inc.), which presented the full dance videos. Each video was shown twice, once for each avatar, in a random order. The dimensions of the dance videos were 640 x 480 mm, which reflected perceptually similar scaling to the physical training condition. Participants listened to the soundtrack that accompanied each sequence via the computer speakers. Participants were instructed to pay close attention to the dance sequences, and were told that they will have to perform the sequences at the end of the week, so they should try to memorize the movements. To test that they were paying close attention, at the end of each music video, ten short dance segments (five from the videos they had just watched) were displayed, without music, each followed by the question ‘Did you see this movement in the video you just watched?’. The participants had to respond ‘yes’ or ‘no’ using the keyboard arrow keys.

***Auditory Experience Only.***

For the sequences that participants received only auditory experience with, they sat at a computer running MATLAB R2010a and Psychophysics Toolbox 3, which presented the two dance video soundtracks twice each in a random order. Visually, participants saw only a black screen and were instructed to listen carefully to the music. To ensure participants paid attention to the music, a short beep was randomly interspersed within the music (10 beeps per sequence) to which the participants had to respond to, using the right arrow of the keyboard. On average, participants responded accurately to 99% of the beeps, with no differences between individual songs.

1. SUPPLEMENTARY RESULTS

2.1 Physical performance over the 4 days of training and VA accuracy

Participants’ physical performance and attention to the VA training procedures improved over the 4 days of training (*F*_1.640, 31.159_ = 68.868, *p* < 0.001 for PVA training, and F_3,57_ = 10.730, *p* < 0.001 for VA training - Supplementary Figure 1). This demonstrates that participants became better at performing the movements from PVA training condition, and at recognizing the constituent movements from the VA training condition, and consequently appeared to become more and more familiar with both the PVA and VA sequences.

Supplementary Figure 1


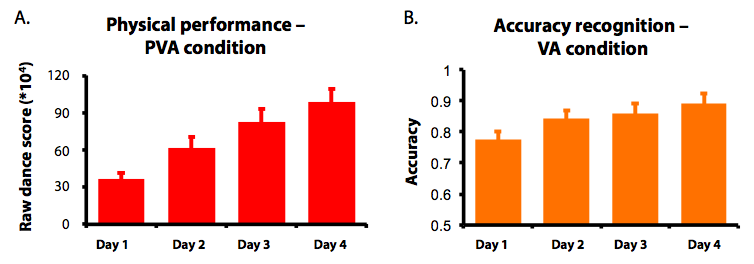


**Supplementary Figure 1. A. Mean dance scores for the PVA trained sequences across training days. Participants’ dance scores significantly improved across the four days of training. B. Mean accuracy recognition task for the VA trained sequences, Participants’ visual recognition of movement segments they watched during VA training significantly improved across the four days of training.**

2.2 Affective judgement – difference between training condition

Pairwise comparisons between training conditions reveal that, post-training, participants rated the PVA sequences as easier to reproduce than sequences from the VA (*p*=0.049), A (*p*<0.001) or UNT (*p*<0.001) conditions; and that only PVA and VA conditions produced a significant different ratings on Day 5 compared to Day 1. VA trained sequences were also considered as easier to reproduce after training than A trained and UNT sequences (*p*=0.010 and *p*=0.041 respectively). Similarly for liking ratings, except that PVA and VA conditions produced similar effect on liking ratings: both training increased significantly the affective judgment of the PVA and VA trained sequences (Figure S1B).
